# Section on Theory and Practice of Medicine

**Published:** 1898-02

**Authors:** A. B. Gardner, Joe S. Wooten

**Affiliations:** Chairman, Bellville, Texas; Sec. Section; Austin, Texas


### Society Notes.

### Section on Theory and Practice of Medicine.

AUSTIN, TEXAS, January 28th, 1898.

DEAR DOCTOR: The State Medical Association meets in Houston on the fourth Tuesday in April. Believing that you will realize the importance of maintaining the program of the "Section on Theory and Practice of Medicine" up to the high standard which in former years it has always occupied, and to a degree commensurate with the time allotted to it; I beg of you to please favor the Association with a paper.

The title of your paper should be sent me as soon as possible, with name of some physician whom you wish to open the discussion, and the paper itself should be in my hands by April 1st, and not later than April 10th. Yours fraternally,

A. B. GARDNER, Chairman, Bellville, Texas.

JOE S. WOOTEN, Sec. Section.

